# Allostatic Load and Racial and Rural Disparities in Breast Cancer Survival

**DOI:** 10.1001/jamanetworkopen.2025.28019

**Published:** 2025-08-21

**Authors:** Yufan Guan, Roger T. Anderson, Supraja Gururaj, Wendy F. Cohn, Philip I-Fon Chow, Bernard F. Fuemmeler, Harry D. Bear, Hua Zhao, Jie Shen

**Affiliations:** 1 Department of Public Health Sciences, School of Medicine, University of Virginia, Charlottesville; 2 Research and Clinical Trial Analytics, School of Medicine, University of Virginia, Charlottesville; 3 Department of Psychiatry and Neurobehavioral Sciences, School of Medicine, University of Virginia, Charlottesville; 4 Department of Family Medicine, School of Medicine, Virginia Commonwealth University, Richmond; 5 Department of Surgery, School of Medicine, Virginia Commonwealth University, Richmond

## Abstract

**Importance:**

Racial and geographic disparities in breast cancer survival persist. Biological consequences of chronic stress, measured by allostatic load (AL), may contribute to these disparities, but their role in breast cancer prognosis is not well understood.

**Objective:**

To evaluate the association between AL and overall survival among patients with breast cancer and assess the contribution of AL to racial and rural disparities in survival.

**Design, Setting, and Participants:**

This cohort study used data from an institutional electronic health record and cancer registry at the University of Virginia Comprehensive Cancer Center. Participants were women diagnosed with stage I to III breast cancer between 2014 and 2024. Data were analyzed from February 2025 through April 2025.

**Exposures:**

Allostatic load was derived from 14 clinical biomarkers and medication history and categorized as low (score ≤3) or high (score >3).

**Main Outcomes and Measures:**

The primary outcome was overall survival. Cox proportional hazards models were used to assess the association with overall survival. Blinder-Oaxaca decomposition was performed to assess the extent to which AL explained disparities in survival by race and rurality.

**Results:**

Among 3069 patients with stage I to III breast cancer followed up for a median (IQR) of 55.4 (30.1-83.3) months (353 Black [11.5%], 2530 White [82.4%], and 178 other race [5.8%]; 1565 aged ≤66 years [51.0%] and 1504 aged >66 years [49.0%]), most individuals were postmenopausal (1523 patients [49.6%]) and married (1766 patients [57.5%]). There was a higher mean (SD) AL score in patients who were older (4.39 [1.68] for ages >66 years vs 3.84 [1.68] for ages ≤66 years), Black (4.54 [1.81] vs 4.07 [1.68] for White patients), rural residents (4.25 [1.72] vs 4.08 [1.70] for urban residents), uninsured (4.35 [1.55]) or on public insurance (eg, 4.33 [1.93] for Medicaid vs 3.70 [1.56] for private insurance), and unemployed (4.14 [1.77]) or retired (4.39 [1.65] vs 3.63 [1.60] for employed) and who reported no alcohol use (4.33 [1.74] vs 3.94 [1.66] for reported use) or a history of tobacco use (4.20 [1.69] vs 4.05 [1.72] for no reported use). High AL score (>3) was associated with an increased risk of mortality (hazard ratios [HRs] ranged from 1.26; 95% CI, 1.04 to 1.54 for model 5 [adjustment for demographics, social economic status, lifestyle factors, and clinical factors] to 1.53; 95% CI, 1.26 to 1.86 for model 1 [crude model]) compared with low AL score (≤3). Stratified analyses indicated a larger HR in the association between AL score and mortality among rural Black patients (HR per 1-unit increase in AL, 3.33; 95% CI, 1.27-8.77). In decomposition analyses, the total explained portion accounted for −0.0708 of the racial disparity, with AL score accounting for −0.0100 (95% CI, −0.0219 to 0.0018), or 14.2% of the explained portion, while the total explained portion of the geographic disparity was −0.0127, with AL score accounting for −0.0043 (95% CI, −0.0107 to 0.0021), or 34.3% of the explained rural disparity, although estimates were not statistically significant.

**Conclusions and Relevance:**

In this study, high AL was independently associated with worse overall survival. The contribution of AL to the observed racial and rural disparities did not reach statistical significance.

## Key Points

**Question:**

Does allostatic load help explain racial and rural disparities in breast cancer survival?

**Findings:**

In this cohort study of 3069 patients with stage I to III breast cancer, higher allostatic load was associated with an increased risk of mortality. Decomposition analyses suggested that the contribution of allostatic load to racial and rural disparities in survival was not statistically significant.

**Meaning:**

This study found that allostatic load was associated with worse breast cancer survival, highlighting the need for interventions that address chronic stress and promote health equity.

## Teaser

This cohort study investigates the association of allostatic load with racial and rural-urban disparities in survival among patients with stage I to III breast cancer.

## Introduction

Breast cancer is the most frequently diagnosed cancer and a leading cause of cancer mortality among women in the US.^1^ While advancements in screening and treatment have improved overall survival, persistent disparities remain in breast cancer outcomes, particularly by race and geographic location.^2,3^ Black women, for instance, are more likely to be diagnosed at later stages and have significantly lower survival rates compared with White women despite similar or lower incidence rates.^4^ Likewise, women living in rural areas often face higher mortality risk compared with their urban counterparts due in part to differences in health care infrastructure, access to care, and social determinants of health.^5,6,7^

These disparities underscore the need to examine upstream factors that may contribute to differential breast cancer outcomes across populations. One such factor is allostatic load (AL), a measure of the cumulative physiological burden imposed by chronic psychosocial and environmental stress.^8,9^ AL reflects the dysregulation of multiple biological systems, including metabolic, cardiovascular, and inflammatory pathways, that can result from prolonged exposure to stressors.^10^ Increased AL has been associated with a range of adverse health outcomes, including premature mortality and increased risk of chronic disease.^9,10^ In the context of breast cancer, chronic stress and the biological sequelae captured by AL may influence tumor biology,^11^ treatment response,^12,13^ and, ultimately, survival.^14,15^

Importantly, AL is not uniformly distributed across populations.^16^ Structural inequalities, including racism, economic disadvantage, and geographic isolation, contribute to greater stress exposure and physiological wear and tear among marginalized groups.^17^ Black individuals and those living in rural areas are disproportionately burdened by factors such as poverty, discrimination, limited access to health care, and social isolation, all of which can contribute to increased AL.^18,19^ These disparities in AL may help explain observed inequities in breast cancer outcomes and offer new insights into the biological embodiment of social disadvantage.

Using patients with breast cancer seen at the University of Virginia Comprehensive Cancer Center in this study, we aimed to determine whether^1^ pretreatment AL was associated with overall survival,^2^ whether this association was moderated by race and urban vs rural residence,^3^ and to what extent AL may explain racial and geographic disparities in breast cancer survival. By integrating biological, social, and geographic dimensions of health, this research sought to elucidate pathways through which chronic stress contributed to inequities in breast cancer survival.

## Methods

### Study Population

In this cohort study, 3069 female patients with breast cancer were identified from a pool of patients with breast cancer seen at the University of Virginia Comprehensive Cancer Center in the last decade (2014-2024). The selection criteria included newly diagnosed stage I to III breast cancer, no prior cancer diagnosis except nonmelanoma skin cancer, and living in the catchment area of the University of Virginia Cancer Center, which includes 3.2 million residents from a large area that includes 87 counties throughout northern, central, southside, and southwestern Virginia, as well as eastern West Virginia. The Strengthening the Reporting of Observational Studies in Epidemiology (STROBE) reporting guideline was followed in the design, analysis, and interpretation of study results. The Institutional Research Board at the University of Virginia approved this study. Informed consent was waived by the institutional review board because this was a retrospective review.

### AL Construction

In this study, AL was assessed using 14 biomarkers routinely collected in clinical practice and commonly referenced in the AL literature.^16,20,21^ Briefly, biomarkers were selected to represent 4 physiological systems: cardiovascular (heart rate, systolic blood pressure, and diastolic blood pressure), metabolic (body mass index [calculated as weight in kilograms divided by height in meters squared] and triglyceride, high-density lipoprotein cholesterol, low-density lipoprotein cholesterol, total cholesterol, alkaline phosphatase, fasting glucose, and albumin levels), kidney (creatinine levels, estimated glomerular filtration rate, and blood urea nitrogen levels), and immune (white blood cell count) systems. In addition to these biomarkers, medication history for diabetes, cardiovascular disease, chronic kidney disease, and hypertension was incorporated into the AL score. To address missing data, multiple imputation by chained equations^22^ was performed to generate 10 imputed datasets. Each biomarker was dichotomized based on established clinical risk thresholds (eTable 1 in Supplement 1), assigning a score of 1 (deemed as high risk) or 0 (deemed as low risk). The AL score was computed as the sum of all positive indicators, yielding a total score ranging from 0 to 15, with higher scores reflecting greater physiological dysregulation. For analytic purposes, AL scores were further categorized into 2 groups: low AL (score ≤3) and high AL (score >3) based on the distribution of scores within the study population.

### Assessment of Covariates

Sociodemographic factors included age, self-reported race (Black, White, and other), menopausal status (premenopausal, perimenopausal, postmenopausal, and other), employment status (employed, unemployed, disabled, and retired), marital status (married and other), insurance status (private, Medicaid, Medicare, military, and uninsured), tobacco use (ever and never), and alcohol consumption (ever and never). Neighborhood characteristics included area deprivation index (ADI)^23^ and Rural-Urban Continuum Codes (RUCC).^24^ ADI was dichotomized into high deprivation (≤5) and low deprivation (>5). RUCC was classified as rural (RUCC >3) or urban (RUCC ≤3). Tumor characteristics included tumor stage and tumor subtype. Tumor stage was classified according to the American Joint Committee on Cancer TNM staging system into stages I, II, and III. Tumors were classified into subtypes, including by estrogen receptor (ER) status and triple-negative breast cancer status, with triple-negative breast cancer tumors defined as lacking expression of ER, progesterone receptor, and human epidermal growth factor receptor 2. Additionally, clinical treatment variables, including chemotherapy and radiation therapy, were each classified as *yes* or *no* based on treatment receipt.

### Outcomes

The primary outcome was overall survival, defined as the time from the date of breast cancer diagnosis to the date of death from any cause. For participants who were alive at the end of the study period, survival time was censored at the date of last known contact or at study completion (September 23, 2024), whichever occurred first. The median (IQR) follow-up for study participants was 55.4 (30.1-83.3) months overall. The median (IQR) follow-up was 60.7 (34.7-86.3) months for patients who were alive and 31.2 (13.6-51.4) months for patients who died.

### Statistical Analysis

Mean differences in AL were evaluated across demographic, lifestyle, clinical, tumor, and socioeconomic characteristics. Poisson regression assessed the joint association of race and geographic location with AL. Associations between AL and breast cancer mortality were examined using Cox proportional hazards models, with sequential adjustments. Model 2 included demographics (age, race, and menopausal status). Model 3 added socioeconomic status (employment, marital, and insurance status). Model 4 included lifestyle factors (tobacco and alcohol use). Model 5 added clinical characteristics (tumor stage and subtype, chemotherapy, and radiation). Model 6 was fully adjusted, incorporating individual- and neighborhood-level factors (area deprivation index and RUCC). Stratified analyses further explored AL-mortality associations within subgroups. To evaluate the contribution of AL to racial and geographic disparities in 3-year breast cancer survival, Blinder-Oaxaca decomposition was applied using separate models for race (Black vs White) and geography (rural vs urban). Models were specified as linear probability regressions and adjusted for key covariates, including age, tumor stage, alcohol use, and triple-negative status. Pooled regression coefficients were used as weights, and 1000 bootstrap replications generated 95% CIs and *P* values for total and variable-specific contributions. All analyses were conducted using R statistical software version 4.3.0 (R Project for Statistical Computing), with 2-sided tests and significance defined as *P* < .05. Data were analyzed from February 2025 through April 2025.

## Results

The study included 3069 patients with breast cancer (353 Black [11.5%], 2530 White [82.4%], and 178 other race [5.8%]; 1565 aged ≤66 years [51.0%] and 1504 aged >66 years [49.0%]), with a mean (SD) AL score of 4.12 (1.70). Most patients were postmenopausal (1523 patients [49.6%]) and married (1766 patients [57.5%]). AL levels varied significantly across demographic subgroups (Table 1). Older patients had a higher mean (SD) AL compared with younger patients (4.39 [1.68] for ages >66 years vs 3.84 [1.68] for ages ≤66 years; *P* < .001). Racial disparities were evident, with Black patients exhibiting the highest mean (SD) AL score (4.54 [1.81]) vs White patients (4.07 [1.68]) and other racial groups (3.89 [1.66]; *P* < .001). Women who were postmenopausal (4.14 [1.67]) and those with other menopausal status (eg, hysterectomy: 4.21 [1.76]) had higher mean (SD) AL scores than women who were premenopausal (3.45 [1.69]; *P* < .001). Socioeconomic status factors were also associated AL, with employed individuals (3.63 [1.60]) showing lower mean (SD) AL scores than unemployed (4.14 [1.77]) or retired (4.39 [1.65]) individuals (*P* < .001) and uninsured (4.35 [1.55]) patients or those with public insurance (eg, 4.33 [1.93] for Medicaid) showing higher mean (SD) AL scores than patients with private insurance (3.70 [1.56]; *P* < .001). Mean (SD) AL score was also higher women in rural areas than those in urban area (4.25 [1.72] vs 4.08 [1.70]; *P* = .04). Lifestyle factors, including tobacco use and alcohol consumption, were also associated with AL levels. Patients who reported ever drinking (3.94 [1.66] vs 4.33 [1.74] for never drinking; *P* < .001) and those who reported never smoking (4.05 [1.72] vs 4.20 [1.69] for ever smoking; *P* = .01) had lower mean (SD) AL scores than their counterparts. In terms of clinical variables, mean (SD) AL score was higher in women diagnosed with ER-positive breast cancer than those with ER-negative breast cancer (4.14 [1.72] vs 3.95 [1.59]; *P* = .02). Notably, patients who died during follow-up had significantly higher mean (SD) AL scores than survivors (4.57 [1.83] vs 4.02 [1.66]; *P* < .001). Next, we explored the joint association of racial and geographic differences with AL among patients with breast cancer (eTable 2 in Supplement 1). Black patients in rural areas had the highest mean AL score (4.70; 95% CI, 4.10-5.30), with an increased relative ratio (1.11; 95% CI, 1.01-1.22) compared with White urban patients. Black urban patients also exhibited increased AL scores (relative ratio, 1.07; 95% CI, 1.01-1.14) compared with White urban patients.

**Table 1.** AL Levels by Patient Characteristics

Characteristic	Patients, No. (%)	AL score, mean (SD)	*P* value
Overall	3069 (100)	4.12 (1.70)	NA
Age group, y			
≤66	1565 (50.99)	3.84 (1.68)	<.001
>66	1504 (49.01)	4.39 (1.68)	
Race			
Black	353 (11.50)	4.54 (1.81)	<.001
White	2530 (82.44)	4.07 (1.68)	
Other^a^	178 (5.80)	3.89 (1.66)	
Menopausal status			
Premenopausal	213 (6.94)	3.45 (1.69)	<.001
Perimenopausal	52 (1.69)	3.52 (1.55)	
Postmenopausal	1523 (49.63)	4.14 (1.67)	
Other^b^	878 (28.61)	4.21 (1.76)	
Marital status			
Married	1766 (57.54)	3.99 (1.70)	<.001
Other^c^	1297 (42.26)	4.28 (1.70)	
Insurance status			
Private	1699 (55.36)	3.70 (1.56)	<.001
Medicare	750 (24.44)	4.42 (1.67)	
Medicaid	138 (4.50)	4.33 (1.93)	
Military	49 (1.60)	4.27 (1.81)	
Uninsured	433 (14.11)	4.35 (1.55)	
Employment status			
Employed	964 (31.41)	3.63 (1.60)	<.001
Unemployed	372 (12.12)	4.14 (1.77)	
Disabled	190 (6.19)	4.41 (1.68)	
Retired	1271 (41.41)	4.39 (1.65)	
ADI within the state of Virginia			
≤5	1633 (53.21)	4.12 (1.70)	.71
>5	1436 (46.79)	4.10 (1.71)	
RUCC			
Urban	2371 (77.26)	4.08 (1.70)	.04
Rural	660 (21.51)	4.25 (1.72)	
Alcohol consumption			
Never	1293 (42.13)	4.33 (1.74)	<.001
Ever	1697 (55.29)	3.94 (1.66)	
Tobacco use			
Never	1762 (57.41)	4.05 (1.72)	.01
Ever	1248 (40.66)	4.20 (1.69)	
Tumor stage			
I	1843 (60.05)	4.10 (1.69)	.60
II	844 (27.50)	4.11 (1.73)	
III	358 (11.64)	4.20 (1.73)	
ER status			
Negative	500 (16.29)	3.95 (1.59)	.02
Positive	2569 (83.71)	4.14 (1.72)	
Triple negative			
No	2789 (90.88)	4.13 (1.72)	.13
Yes	280 (9.12)	3.98 (1.57)	
Chemotherapy treatment			
Yes	1104 (35.97)	4.13 (1.71)	.74
No	1965 (64.03)	4.11 (1.70)	
Radiation treatment			
Yes	1589 (51.78)	4.11 (1.73)	.93
No	1480 (48.22)	4.12 (1.67)	
Death			
Yes	494 (16.10)	4.57 (1.83)	<.001
No	2537 (82.67)	4.02 (1.66)	

Abbreviations: ADI, area deprivation index; AL, allostatic load; ER, estrogen receptor; NA, not applicable; RUCC, Rural-Urban Continuum Codes.

^a^ Other race includes Asian, Native Hawaiian, or Other Pacific Islander and multiracial (ie, >1 race category) individuals.

^b^ Other menopausal status includes ablation, hysterectomy, and other.

^c^ Other marital status includes divorced, legally separated, single, widowed, other, and significant other.

**Figure 1.**
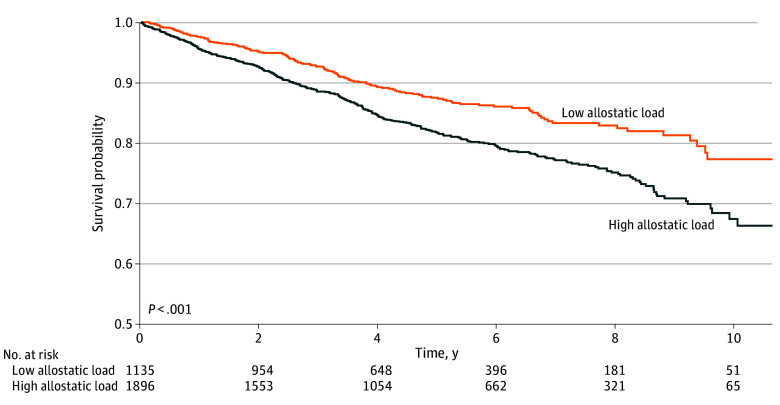
Kaplan-Meier Survival Estimates for the Association Between Allostatic Load Score and Breast Cancer Mortality Allostatic load was categorized as low (score ≤3) or high (score >3).

We next assessed the association between AL and breast cancer overall survival. Kaplan-Meier curves (Figure 1) demonstrated a survival disadvantage for patients with high AL (>3) vs low AL (≤3) scores (log-rank *P* < .001). The divergence in survival probabilities emerged early and widened over time. Higher AL was consistently associated with increased mortality risk across all adjusted models (Table 2). In the crude model (model 1), AL score as a continuous variable was associated with an increased hazard of death (HR per 1-unit increase in AL score, 1.19; 95% CI, 1.13-1.25). This association persisted after sequential adjustment for demographics (model 2), socioeconomic status (model 3), lifestyle factors (model 4), clinical variables (model 5), and neighborhood characteristics (model 6: HR, 1.14; 95% CI, 1.08-1.21; *P* < .001). In a sensitivity analysis among only 2332 patients with complete biomarker data, the HR for death per 1-unit increase in AL score was 1.16 (95% CI, 1.08-1.24; *P* < .001). When AL score was dichotomized as high (>3) vs low (≤3), high AL was associated with increased mortality risk across models, with HRs ranging from 1.26 (95% CI, 1.04-1.54) for model 5 to 1.53 (95% CI, 1.26-1.86) for model 1. In model 6, the HR was 1.27 (95% CI, 1.04-1.55). Additionally, we conducted a sensitivity analysis using AL quartiles. Results from these quartile-based analyses were consistent with our primary findings (eTable 3 in Supplement 1).

**Table 2.** Association Between AL and Overall Survival

AL score	Death, HR (95% CI)					
	Model 1^a^	Model 2^b^	Model 3^c^	Model 4^d^	Model 5^e^	Model 6^f^
Continuous, per 1-unit increase	1.19 (1.13-1.25)	1.17 (1.11-1.23)	1.14 (1.08-1.20)	1.13 (1.07-1.19)	1.14 (1.08-1.20)	1.14 (1.08-1.21)
Categorical						
Low (≤3)	1 [Reference]	1 [Reference]	1 [Reference]	1 [Reference]	1 [Reference]	1 [Reference]
High (>3)	1.53 (1.26-1.86)	1.42 (1.17-1.74)	1.29 (1.06-1.58)	1.28 (1.05-1.57)	1.26 (1.04-1.54)	1.27 (1.04-1.55)

Abbreviation: AL, allostatic load; HR, hazard ratio.

^a^ Crude model.

^b^ Model 1 plus demographics (including age, race, and menopausal status).

^c^ Model 2 plus social economic status (including employment status, marital status, and insurance status).

^d^ Model 3 plus lifestyle factors (including alcohol use and tobacco use).

^e^ Model 4 plus clinical factors (including tumor stage, estrogen receptor status, triple-negative status, chemotherapy, and radiation treatment).

^f^ Model 5 plus neighborhood variables (including area deprivation index and Rural-Urban Continuum Codes).

**Figure 2.**
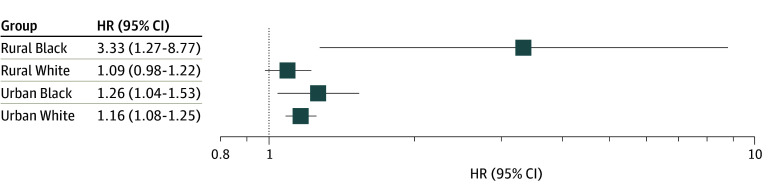
Association Between Allostatic Load and Breast Cancer Mortality by Race and Rurality HR indicates hazard ratio.

We further investigated whether the risk association between AL and breast cancer survival differed by race and urban vs rural residence. The associated increase in risk was higher among Black (HR, 1.18; 95% CI, 1.01-1.39) than White (HR, 1.15; 95% CI, 1.08-1.21) patients with breast cancer. For urban vs rural residence, the increase in risk was significant among patients with urban residence (HR, 1.16; 95% CI, 1.09-1.23) but not among those with rural residence (HR, 1.10; 95% CI, 0.99-1.22). When we combined race and urban vs rural residence (Figure 2), racial and geographic factors interacted with the risk association between and AL and breast cancer survival. Per 1-unit increase in AL, the hazard of death increased among urban White (HR, 1.16; 95% CI, 1.08-1.25), urban Black (HR, 1.26; 95% CI, 1.04,-1.53), and rural White (HR, 1.09; 95% CI, 0.98-1.22) patients. Notably, rural Black women exhibited a substantially increased hazard of death (HR, 3.33; 95% CI, 1.27-8.77) per unit increase in AL score. We further assessed interactions among race, rural residence, and AL score; however, none reached statistical significance.

Finally, we performed Blinder-Oaxaca decomposition to evaluate to what extent AL may explain racial and geographic disparities in breast cancer survival (Table 3). Among Black and White patients, the total disparity was −0.0148, reflecting worse outcomes among Black patients. The explained portion accounted for −0.0708 of the disparity, while the unexplained component (0.0560) offset part of the gap. Allostatic load alone explained −0.0100 (95% CI, −0.0219 to 0.0018), or 14.2% of the explained disparity; although its contribution was not statistically significant (*P* = .11), it ranked as the third-largest contributor after tumor stage (−0.0270; 95% CI, −0.0470 to −0.0070 [38.1%]; *P* = .01) and triple-negative breast cancer (−0.0223; 95% CI, −0.0423 to −0.0033 [31.5%]; *P* = .02). In the rural-urban comparison, the total disparity was larger (−0.0304), with observed covariates accounting for −0.0127 of the disparity (41.8%) and −0.0177 of the disparity (58.2%) attributed to unexplained factors. AL emerged as one of the leading contributors to the explained rural-urban disparity, accounting for −0.0043 (95% CI, −0.0107 to 0.0021), or 34.3% of the explained disparity. Although this contribution was not statistically significant (*P* = .17), it exceeded that of tumor stage (−0.0042; 95% CI, −0.0164 to −0.0011 [33.2%]; *P* = .02) and age (−0.0021; 95% CI, −0.0083 to 0.0041 [16.6%]; *P* = .51).

**Table 3.** Blinder-Oaxaca Decomposition Analyses of Role of AL in Mortality Disparities

Variable	Estimated mean		Proportion of explained disparity	Proportion of total explained, %	*P* value
	Comparison group	Reference group			
**Racial disparity** ^a^					
AL	4.49	4.07	−0.0100 (−0.0219 to 0.0018)	14.20	.11
Age	64.77	67.04	−0.0004 (−0.0103 to 0.0094)	0.60	.93
Stage	0.19	0.11	−0.0270 (−0.0470 to −0.0070)	38.10	.01
Alcohol use	0.38	0.58	−0.0150 (−0.0337 to 0.0044)	21.10	.10
TNBC	0.21	0.08	−0.0223 (−0.0423 to −0.0033)	31.50	.02
Rurality	0.28	0.22	0.0040 (−0.0036 to 0.0116)	−5.70	.33
Total explained	NA	NA	−0.0708	100	NA
Unexplained	NA	NA	0.0560	NA	NA
Total disparity	NA	NA	−0.0148	NA	NA
**Geographic disparity** ^b^					
AL	4.27	4.08	−0.0043 (−0.0107 to 0.0021)	34.25	.17
Age	67.31	66.58	−0.0021 (−0.0083 to 0.0041)	16.63	.51
Stage	0.13	0.12	−0.0042 (−0.0164 to −0.0011)	33.19	.02
Alcohol use	0.46	0.58	−0.0054 (−0.0164 to 0.0057)	42.40	.35
TNBC	0.11	0.10	−0.0009 (−0.0034 to 0.0014)	6.79	.39
Race	0.16	0.12	0.0042 (−0.0036 to 0.0120)	−33.26	.33
Total explained	NA	NA	−0.0127	100	NA
Unexplained	NA	NA	−0.0177	NA	NA
Total disparity	NA	NA	−0.0304	NA	NA

Abbreviations: AL, allostatic load; NA, not applicable; TNBC, triple-negative breast cancer.

^a^ Racial disparity is between Black (comparison group) and White (reference group) patients.

^b^ Geographic disparity is between rural (comparison group) and urban (reference group) patients.

## Discussion

In this cohort study of more than 3000 patients with breast cancer treated at the University of Virginia Comprehensive Cancer Center, increased AL, a marker of cumulative physiological stress, was associated with decreased overall survival. However, the magnitude of HRs in this association varied across population subgroups. Black women and those residing in rural areas had significantly higher AL scores than their White and urban counterparts. While increased AL was associated with worse survival across the cohort, HRs were highest among Black women regardless of geographic location. Rural Black women had the highest AL levels, suggesting greater cumulative stress exposure, and rural Black women exhibited the highest HR in the association between AL and mortality risk. These patterns indicate that both race and geographic context may interact with the association between AL and survival, potentially reflecting differences in structural stressors, access to care, or tumor biology. Further research is warranted to disentangle these intersecting factors and their roles in breast cancer disparities.

We found that AL varied significantly across demographic and socioeconomic subgroups. Black patients, those living in rural areas, and individuals with lower socioeconomic status exhibited higher AL scores, consistent with existing literature showing that marginalized populations are disproportionately exposed to chronic stressors.^18,19,25^ These stressors, rooted in systemic racism, poverty, and geographic isolation, contribute to the dysregulation of multiple physiological systems. Our observation that Black women, particularly those living in rural areas, had the highest AL scores underscores the intersectional nature of structural disadvantage and its physiological consequences.

The survival analysis revealed that higher AL score was associated with increased mortality risk. Patients with high AL scores had significantly worse survival outcomes, with each unit increase in AL corresponding to an increase in the hazard of death across adjusted models, with HRs ranging from 1.13 to 1.19. This dose-response pattern is consistent with the hypothesis that AL captures cumulative biological wear and tear that may impair the body’s capacity to cope with cancer and its treatment. Our results are consistent with previous reports by Obeng-Gyasi et al,^14,15^ in which they found a similar association between increased AL and all-cause mortality in patients with breast cancer. The association between AL and poor outcomes may reflect stress-related disruptions in immune function, inflammation, endocrine regulation, and metabolic balance, all of which can influence tumor progression, treatment resistance, and recovery.^26,27^ Notably, the association between AL and mortality varied by race and geographic location. Stratified and joint effect analyses revealed that rural Black women experienced the highest mortality risk associated with increased AL, followed by urban Black and urban White women. Rural Black women exhibited a markedly increased risk per 1-unit increase in AL of death (HR, 3.33), compared with a smaller increase among urban White women (HR, 1.16), emphasizing the complex and multifactorial nature of racial and geographic disparities in breast cancer outcomes.^28^

In this study, we applied the Blinder-Oaxaca decomposition method to examine factors contributing to racial and rural disparities in breast cancer outcomes, with a particular focus on AL as a potential explanatory factor. While AL did not reach statistical significance, it accounted for a meaningful portion of the explained disparity: 14.2% in racial comparisons and 34.3% in rural-urban comparisons. These contributions were on par with or exceeded those of more commonly studied factors, such as age and tumor stage. However, these findings should be interpreted cautiously. The lack of statistical significance may, in part, reflect limited sample sizes; only 11.5% of the study population identified as Black, and 21.5% of patients resided in rural areas, thereby reducing power to detect robust associations. Nonetheless, these results are consistent with prior research associating chronic stress with adverse breast cancer outcomes, especially in socially and structurally marginalized populations.^29,30^ Although exploratory, these findings point to the potential biological relevance of AL in cancer disparities and underscore the need for further research in more diverse and adequately powered cohorts.

### Strengths and Limitations

Despite the strengths of this study, including its large, regionally diverse sample and comprehensive adjustment for confounders, several limitations must be acknowledged. AL was measured using available clinical biomarkers, which represent a clinically feasible AL measure for retrospective electronic health record–based studies and may not capture the full breadth of physiological stress responses, such as neuroendocrine markers. Residual confounding due to unmeasured stressors, such as discrimination, trauma, or caregiving burden, may have also influenced our estimates. In addition, while multiple imputation was used to address missing data, measurement error in self-reported variables could introduce bias. To address this concern, we conducted a sensitivity analysis restricted to patients with breast cancer with complete biomarker data. The analysis revealed a statistically significant increase in the hazard of death per 1-unit increase in AL score (HR, 1.16; 95% CI, 1.08-1.24, *P* < .001) after adjusting for all covariates. Additionally, as an observational study, this study cannot establish causality. We cannot rule out reverse causality (eg, worse cancer prognosis increasing AL). Thus, our findings should be interpreted within the context of a regional cancer center that may not reflect other health care settings.

## Conclusions

This cohort study found that increased AL was associated with shorter breast cancer mortality, and our findings may contribute to understanding persistent racial and rural disparities in survival outcomes. While contributions of AL to these disparities did not reach statistical significance, observed patterns suggest that chronic stress and physiological dysregulation may play a supportive role in shaping inequities. These findings underscore the potential value of integrating biological measures of stress into cancer disparities research, alongside social determinants of health. Future studies with larger and more diverse populations are needed to clarify the role of AL in cancer outcomes. In the meantime, addressing chronic stress and promoting supportive care strategies may hold promise for improving survivorship, especially among Black women and rural patients who may experience disproportionate stress burdens.

## Notes

Supplement 1. **eTable 1.** Distribution and high-risk thresholds of individual biomarkers for allostatic load **eTable 2.** AL levels by racial and geographic differences among patients with breast cancer **eTable 3.** Sensitivity analysis to assess the association between AL and overall survival among patients with breast cancer

Supplement 2.
Data Sharing Statement
