# Supplementary material for: Allostatic Load and Racial and Rural Disparities in Breast Cancer Survival
Source: JAMA Netw Open. 2025 Aug 21;8(8):e2528019. doi: 10.1001/jamanetworkopen.2025.28019 (PMC12371513; doi:10.1001/jamanetworkopen.2025.28019)
Supplement: Supplement 1. — eTable 1. Distribution and high-risk thresholds of individual biomarkers for allostatic load eTable 2. AL levels by racial and geographic differences among patients with breast cancer eTable 3. Sensitivity analysis to assess the association between AL and overall survival among patients with breast cancer [file jamanetwopen-e2528019-s001.pdf]

Supplemental Online Content

Guan Y, Anderson RT, Gururaj S, et al. Allostatic load and racial and rural disparities in breast cancer survival. *JAMA Netw Open*. 2025;8(8):e2528019. doi:10.1001/jamanetworkopen.2025.28019

eTable 1. Distribution and high-risk thresholds of individual biomarkers for allostatic load

eTable 2. AL levels by racial and geographic differences among patients with breast cancer

eTable 3. Sensitivity analysis to assess the association between AL and overall survival among patients with breast cancer

This supplemental material has been provided by the authors to give readers additional information about their work.

© 2025 Guan Y et al. *JAMA Network Open*.

eTable 1. Distribution and High-Risk Thresholds of Individual Biomarkers for Allostatic Load

		Initial(N=3069)			After Imputation(N=3069)	
	Cutoff Value	at Risk	% at Risk	% missing	at Risk	% at Risk
SBP-mmHg	≥140	743	24.21	19.52	916	29.85
DBP-mmHg	≥90	107	3.49	19.52	131	4.27
Pulse	>=100	137	4.46	20.10	172	5.60
BMI	>=25	1713	55.82	20.56	2141	69.76
Fast-gluc-mg/dl	>=126	407	13.26	23.98	547	17.82
Albumin-g/dl	>5.5 or <3.5	79	2.57	26.69	112	3.65
Creatinine-mg/dl	>1.1 or <0.6	258	8.41	23.88	333	10.85
eGFR-ml/min	<90	1564	50.96	23.88	1993	64.94
WBC-k/ul	>11 or <4.5	351	11.44	24.01	461	15.02
BNU-mg/dl	>20 or <7	353	11.50	24.37	443	14.43
ALP-IU/l	>147 or <44	110	3.58	26.72	156	5.08
LDL-mg/dl	>=130	414	13.49	60.48	1098	35.78
Total Chol-mg/dl	>=240	160	5.21	60.64	395	12.87
HDL-mg/dl	<=50	521	16.98	60.41	1333	43.43
Trig-mg/dl	>=150	336	10.95	60.28	846	27.57
Medical history	Yes	1924	62.69		1924	62.69

© 2025 Guan Y et al. *JAMA Network Open*.

eTable 2. Al levels by racial and geographic differences among breast cancer patients

	AL, Mean (95%CI)		Adjusted relative ratio (95%CI)*
	Crude	Adjusted*	
White-Urban	4.05 (3.97, 4.12)	4.22 (3.71, 4.74)	Reference
White-Rural	4.15 (4.00, 4.29)	4.23 (3.70, 4.75)	1.01 (0.95, 1.05)
Black-Urban	4.45 (4.22, 4.67)	4.51 (3.96, 5.06)	1.07 (1.01, 1.14)
Black-Rural	4.76 (4.42, 5.10)	4.70 (4.10, 5.30)	1.11 (1.01, 1.22)

*. Poisson regression Adjusted with age, menopausal status, marital status, insurance type, employment, ADI, alcohol, tobacco, and stage.

© 2025 Guan Y et al. *JAMA Network Open*.

eTable 3. Sensitivity analysis to assess the association between AL and overall survival among breast cancer patients

	Model 1	Model 2	Model 3	Model 4	Model 5	Model 6
AL (categorical)						
Q1	Reference	Reference	Reference	Reference	Reference	Reference
Q2	1.27 (0.99, 1.62)	1.22 (0.95, 1.56)	1.09 (0.86, 1.41)	1.08 (0.84, 1.38)	1.01 (0.78, 1.28)	1.01 (0.78, 1.28)
Q3	1.24 (0.94, 1.63)	1.16 (0.88, 1.53)	1.07 (0.81,1.41)	1.07 (0.81, 1.41)	1.10 (0.83, 1.45)	1.09 (0.83, 1.44)
Q4	**2.14 (1.70, 2.69)**	**1.95 (1.54, 2.46)**	**1.75 (1.38, 2.21)**	**1.71 (1.35, 2.16)**	**1.78 (1.40, 2.25)**	**1.79 (1.42, 2.27)**
P for Trend	<0.01	<0.01	<0.01	<0.01	<0.01	<0.01

Model 1: Crude model

Model 2: Model 1 + demographics (including age, race, and menopausal status)

Model 3: Model 2 + social economic status (including employment status, marital status, and insurance status)

Model 4: Model 3+ lifestyle factors (including alcohol use and tobacco use)

Model 5: Model 4+clinical factors (including tumor stage, ER status, Tri-negative status, chemotherapy and radiation treatment)

Model 6: Model 5+ neighborhood variables (including ADI and RUCC)
