# Supplementary material for: Allostatic Load and Racial and Rural Disparities in Breast Cancer Survival
Source: JAMA Netw Open. 2025 Aug 21;8(8):e2528019. doi: 10.1001/jamanetworkopen.2025.28019 (PMC12371513; doi:10.1001/jamanetworkopen.2025.28019)
Supplement: Supplement 2. — Data Sharing Statement [file jamanetwopen-e2528019-s002.pdf]

Data Sharing Statement

Guan. Allostatic Load and Racial and Rural Disparities in Breast Cancer Survival. *JAMA Netw Open*. Published online August 21, 2025. doi:10.1001/jamanetworkopen.2025.28019

Data
Data available: No

Additional Information
Explanation for why data not available: Data requests should be directed toward the University of Virginia Office of Sponsored Program. To gain access to the data requesters will need to sign a data access agreement with the University of Virginia.
